# Morbidity at elementary school entry differs by sex and level of residence urbanization: a comparative cross-sectional study

**DOI:** 10.1186/1471-2458-7-358

**Published:** 2007-12-25

**Authors:** Rea-Jeng Yang, Jiunn-Jye Sheu, Huey-Shys Chen, Kuan-Chia Lin, Hsiu-Li Huang

**Affiliations:** 1 Department of Nursing, National Taipei College of Nursing, Pei-Tou, 11219 Taipei City, Taiwan; 2 Department of Health Education and Behavior, University of Florida, Gainesville, Florida, 32611-8210, USA; 3 School of Nursing, University of Medicine and Dentistry of New Jersey, Newark, New Jersey, 07107-3001, USA

## Abstract

**Background:**

Health is vital to a child's learning in school and success in life. Therefore, early physical examination, and follow-up if necessary, would bring parents' attention to their child's health and would likely improve outcomes. The purposes of this study are twofold: to assess the health status of first-graders and to examine the health status differences between sexes, levels of residence urbanization, and quantity of available medical resources.

**Methods:**

This is a comparative descriptive study. Data from the 2002 Student Entry Physical Examination (SEPE) and Student Medical History Inventory (SMHI) were obtained from 203 public and private elementary schools in northern Taiwan where a population of 53,053 students was included. Frequencies, independent sample t test, one-way ANOVA along with Scheff's post hoc test, and Pearson's correlation were conducted using SPSS.

**Results:**

This study showed that 13.7% of students had at least one diagnosed disease from the SMHI reported by parents. Moreover, the SEPE indicated that 79.5% students had at least one health concern. Dental caries, myopia, and obesity were the most prevalent health problems among the first-graders (69.6%, 27.1%, and 9.5%, respectively). Research results show that there were significant differences in the prevalence of dental caries, myopia, and obesity between different sexes and among levels of urbanization. However, the quantity of available medical resources made no significant difference.

**Conclusion:**

Elementary school entry physical examination is an important way to detect students' health problems. It is suggested that school health interventions consider students' health profiles along with their sex and level of urbanization in planning. More research is needed to find the risk factors of the health problems. Additionally, the creation of a school health committee is suggested to implement and evaluate the entry health examination program.

## Background

Health is vital to a child's ability to learn and to succeed in life [1]. Diseases or health problems can prevent a child from fully engaged in learning activities. For instance, studies found that childhood asthma is associated with an increased risk for school problems, including grade failure and learning disability [2,3]. As a means of primary prevention, many studies suggested that physical examination can identify high risk groups among school-aged children and also provide clues for secondary prevention [4,5]. Physical examination is essential in the school health service component of many school health models, such as the Three-Component model, ACCESS model, the Illinois Department of Health model [6], and the recent Health Promoting School model [7]. School entry physical examination has a positive effect upon the high-risk population regarding health, which reduced death from cerebrovascular disease (CVD), heart disease, and so on [5]. Thus, some schools require physical examination before enrollment or sport activities [8].

In Fry's opinions [9], vision, hearing, and growth should be required in school entry examinations "because it may be only the first time that anyone has looked for any abnormality in these areas." The meta-analysis by Ni Bhrolchain [10] reviewed nine British studies and found 55–132 health problems per 100 children examined, while 28%–75% of them were first identified in routine school entry medical examination. This comparison of routine and selected school entry medical examinations also revealed that, if financially supported, the routine examinations had higher percentages of health problems detected and first identified health problems, more referrals, and better identification of undescended testes and speech delay. Another more comprehensive review also reported more detected health problems and elevated percentages of first identified health problems among elementary school entrants [4].

The benefits of physical examination for children, if financially supported, are essentially fourfold: First of all, some impairments and physical changes associated with diseases, which parents may be unaware of due to limited medical knowledge, can cause significant disability or handicap. Thus physical examination may raise parents' awareness of potential health problems in their children. Parents' lack of knowledge may also cause health problems to go undiagnosed in childhood. Secondly, early diagnosis followed by effective treatment is likely to improve disease outcomes. Thirdly, physical examination identifies unmet health needs before any negative health impacts occur and provides an effective health check for children who start school with health and developmental disadvantages [11]. Fourthly, health problems ignored or unknown can be identified by clinicians. From a detailed review of school health records, Elliott and colleagues found 45% of children had at least one problem not previously noted, including undescended testes, heart murmur, squints, hernias, and et al. [12].

Reduced visual acuity may adversely affect sporting and academic achievement in school. The American Academy of Pediatrics (AAP) and the Canadian Task Force on the Periodic Health Examination recommended that clinicians perform objective vision screening on school-aged children. Moreover, both the American Academy of Ophthalmology (AAO) and the American Association for Pediatric Ophthalmology and Strabismus (AAPOS) recommended that screening after five years of age be carried out during routine school checks [13]. There is no doubt that the entry into elementary school is a good time for physical examination.

In addition to the vision screening recommendations by the professional organizations to support health examination from early age, research show health problems in childhood are highly associated with adulthood diseases. For example, obese children have an elevated risk of adulthood high blood pressure, non-insulin-dependent diabetes, high triglyceride/cholesterol circulating levels, and fatty liver than non-obese children [14]. The strong association between childhood obesity and adult diseases shows the importance of early detection and suggests that prevention and treatment of childhood obesity should be pursued to reduce the morbidity and mortality [15]. Physical examination from the elementary school ages would provide a good track of the physical development and health problems, which can provide parents, schools, and health professionals more information in prevention and treatment. Other examples such as early detection of developmental dysplasia of the hip, dental caries, growth disorders, or myopia would allow prompt surgery, treatment, correction, or rehabilitation as needed before deterioration.

Many studies reported morbidity and prevalence of diseases among school-aged children. These studies, however, focus mostly on one specific health problem (i.e., learning disability, vision, and hearing) considering other potential health problems. Nevertheless, the lack of epidemiological studies which examined students' comprehensive physical health status at the entry point of elementary school creates a problem in that no baseline data is available in assessing and evaluating students' overall health in school. The absence of reliable data have made monitoring the health status of children a challenge [16] and have made it difficult to manage the extent of public health problems. Through the elementary school entry physical examination, children's health condition can be established, which would help school nurses manage chronic conditions and the school to conduct more effective school health programs. Specifically, free comprehensive physical examinations would eliminate the financial barriers that accompany different levels of urbanization and available medical resources.

Taipei County is the largest county in Taiwan: with a population of 3,641,446 people, Taipei County comprised 16.2% of the total population of Taiwan in 2002 [17]. Mandatory education is required from the first to ninth grade. To achieve the goals of healthy schools with healthy students, school health promotion programs were launched in Taipei County. Due to the necessity of having baseline data from physical examination at school entry the county offered free comprehensive physical examinations every fall for first graders, the youngest students entering elementary school in the Taiwan education system.

Health and education professionals need information about the prevalence of health problems and their influential factors to develop effective and targeted interventions to reduce health problems in childhood. By reviewing individual disease prevalence reported, students' gender and level of residence urbanization seem to relate to morbidity. From the literature, school children's vision abnormality differed by sex [18], while asthma [19], dental caries [20], and vision screening results differed by urbanization [18-21]. Besides, in Leff's opinion [22], "school health varies dramatically between inner city, urban, and rural schools." Another study, which found the care needs in schools serving disadvantaged areas to be eight times greater than in other areas, seems to imply the difference is due to residency [23]. However, the relationships between health status and number of medical resources have rarely been investigated and are still unclear.

Therefore, the aim of this study was to explore the health status of first-graders in Taipei County, identify the top three health problems, and examine the differences between sexes, levels of urbanization, and the quantity of medical resources. This free physical examination was also intended to preliminarily screen health problems so parents could take their children for detailed diagnosis and treatment if necessary.

## Methods

### Subject

The data used in this comparative descriptive study were collected in the fall of 2002 from the first-graders of all public elementary schools in Taipei County, Taiwan. More than fifty-three thousand (53,053 out of 53,642, 98.9%) students from 203 (out of 211, 96.2%) schools were included in the study. The study was reviewed and approved by the Taipei County Government Education Bureau. Informed written consent was sent to and permission was obtained from the students' parents or guardians. The parents or guardians were required to accompany their children to the physical examination. Students or parents/guardians were free to withdraw from any examination item at any time. The consent form, offered by the Taiwan County Education Bureau, informed parents/guardians that these examinations are non-intrusive with minimum risk. In addition, the students were notified to wear suitable clothing for physical examination.

### Measurement

Data from the 2002 Student Entry Physical Examination (SEPE) and Student Medical History Inventory (SMHI) were analyzed. SEPE assessed eyes, teeth, ears-nose-throat (ENT), heart-lung-abdomen (HLA), bones-muscle, reproductive-urinary, skin and other systems grouped by the regulations set by the Taiwan Ministry of Education [24]. The ICD-9 was used as diagnostic criteria. SMHI, a structural checklist to investigate students' medical history, was filled out by parents/guardians.

Density of the population is the major indicator for the urbanization level in Taiwan. In this study, the levels of urbanization were categorized into 3 groups, urban (above ten thousand), suburban (ranged one thousand to below ten thousand), and rural (below one thousand) based on density of the population (person per square kilometer, km^2^). Based on this categorization, the distribution of schools was 31.5% urban (n = 64), 49.3% suburban (n = 100), and 19.2% rural (n = 39). The quantity of available medical resources, defined by the number of health care facilities, excluding home care, long-term care, hospice, psychiatric, OB-GYN, and EMS facilities, was obtained from the National Health Insurance Office. The quantity of available medical resources ranged from 1 to 397 (mean = 104.6, SD = 109.8, median = 69.0) and varied by school's township. Because national health insurance is obligatory for Taiwan citizens, the registered health care providers represent the most accurate number of available health care facilities.

### Procedure

During the fall semester of 2002, parents/guardians provided consented for their children to participate in the physical examination. A physical examination team including pediatricians, dentists, oculists, and nurses was employed to conduct physical examinations in the health center of each school. In the meantime, the students' parents/guardians were asked to complete and return the SMHI to their children's school.

### Data Analysis

Statistical analyses were carried out using the statistical package SPSS. All data were entered, summarized, and analyzed. Frequencies, independent sample t-test, and analysis of variance (ANOVA) along with post hoc tests were used to examine prevalence of health problems between sexes and levels of urbanization while Pearson's correlation coefficients were used to determine its association with the quantity of medical resources.

## Results

This study explored the morbidity of health problems among first-graders and its association with sex, levels of urbanization, and quantity of medical resources. Of the 53,053 first-grade students aged six to eight years, 52% were boys (27,359) and 48% were girls (25,694). The morbidity of each physical system is represented by cases per thousand while the prevalence of individual disease is shown by percentage.

### Results from Student Medical History Inventory (SMHI)

Results showed 13.7% of students had a medical history of at least one diagnosed illness. The common medical conditions were ranked as follows: allergy (6.7%), asthma (4.1%), hernia (1.5%), Glucose-6-phosphate dehydrogenase deficiency (G6PD, 1.3%), heart disease (0.8%), renal disease (0.6%), tuberculosis (0.6%), hepatitis (0.4%), and epilepsy (0.3%).

### Results from Student Entry Physical Examination (SEPE)

Results showed 79.5% of students had at least one health problem and, on average, each student had 1.6 health problems. Table 1 and Table 2 contain the morbidity of the physical systems and the prevalence of the most frequent health problems respectively. The disease prevalence among boys and girls and reported by system examined.

**Table 1:** Morbidity of physical systems among first-graders (N = 53,053; Boy = 27,359, Girl = 25,694)

Physical system	Sum	Boy	Girl Significance
Eyes	47.8	46.6	49.2***
Teeth	99.8	100.5	99.0***
Ears, Nose, Throat	3.1	3.5	2.7***
Heart, Lung, Abdomen	2.0	2.1	2.2*
Bones, Muscle	0.8	0.9	0.6***
Reproductive	2.2	3.4	0.8^@^
Skin	0.6	0.6	0.6***

Total	159.9	161.5	158.2***

Unit: per 100 people

Statistically significant differences between boys and girls: * indicates p < .05; *** indicates p < .001.

^@^indicates not comparable between boys and girls.

**Table 2:** Prevalence rates of the most common health problems among first-graders (N = 53,053; Boy = 27,359, Girl = 25,694)

Health problem	Overall	Boy	Girl Significance
Myopia	27.1	25.8	28.5***
Caries	69.6	70.0	69.1***
Obesity	9.5	10.6	8.4***
Dysarthria	2.5	3.0	2.0***
Cryptorchidism	-	2.1	-
Heart murmur	1.5	1.6	1.5
Scoliosis	0.4	0.4	0.4
Atopic dermatitis	0.2	0.2	0.2

Statistically significant differences between boys and girls: ***indicates p < .001.

#### Eyes

Morbidity of eyes (composed of myopia, hyperopia, amblyopia, strabismus, astigmatism, color-blindness, nystagmus, parachromatism, and ptosis) was 47.8% (boys 46.6%, girls 49.2%). Girls were found to have more significant morbidity of eye problems than boys (*p *< .001). Myopia (defined as spherical equivalents equal to or less than -0.5 D) was the most serious problem with an overall prevalence of 27.1%. Girls also had higher significant myopia prevalence (28.5%) than boys (25.8%, *p *< .001).

#### Teeth

The teeth problems examined included caries, dental calculus, gingivitis, and periodontal disease. The findings showed that the morbidity of dental problems was 99.8%. That is, almost every first-grader had one teeth problem. Boys (100.5%) had higher significant morbidity than girls (99.0%, *p *< .001). Amongst the dental problems examined, untreated caries was the most popular problem in the first-graders (69.6%). Boys (70.0%) had higher caries prevalence than girls (69.1%) (*p *< .001).

#### ENT system

The problems in the ENT system examined included hearing impairment, tympanic membrane perforation, dysarthria, and lip cleft and palate. The morbidity of the ENT system was 3.1%. Boys had higher significant morbidity (3.5%) than girls (2.7%) (*p *< .001). The most common ENT problem was dysarthria. Boys had a higher prevalence rate as well (3.0% vs. 2.0%, p < .001).

#### HLA system

The health problems examined in this system were composed of arrhythmia, heart murmur, asthma, and hepatomegalia. On average, the morbidity approximated 2.0%. Heart murmur was the most common condition (1.5%) of the HLA system. In addition, 0.2% of first-graders were found to have asthma (0.2% for boys and 0.1% for girls).

#### Bones and muscle system

Poliomyelitis, scoliosis, torticollis, kyphosis, lordosis, and arthrosis derformans were examined in this system. The morbidity of this system was 0.8%. The most common problem was scoliosis (0.4%). No difference in sexes was identified.

#### Reproductive-urinary system

Items examined in the reproductive-urinary system included hernia for both boys and girls except hernia. Boys were also examined for cryptorchidism and scrotal hydroceles. The boys' most common problem was cryptorchidism (2.1%). This system's morbidity was 3.4% for boys and 0.8% for girls.

#### Skin system

Hemangioma, dermatitis, infection, head-lice, tinea, warts, purpura, and scabes were inspected in the skin system. Morbidity of the skin system was 0.6%, and atopic dermatitis (0.2%) was the most common problem found.

#### Other systems

The items examined in the other systems included body height, body weight, urine protein, and parasitism. The most common problem was obesity (defined by the Taiwan Ministry of Education [19] as body weight greater than 20% of ideal weight based on BMI). Obesity prevalence was 9.5%, with boys (10.6%) being significantly more obese than girls (8.4%) (*p *< .001).

### Three top health problems among levels of urbanization

As presented in Table 2, untreated dental caries, myopia, and obesity were the most prevalent health problems among the first-graders (69.6%, 27.1%, and 9.5%, respectively). To find out if the top three health problems varied by level of urbanization, one-way ANOVA along with Scheffe's post hoc comparisons were conducted. Table 3 indicates that the level of urbanization had a significant association with myopia (*F*_(2,199) _= 6.1, p < .01) and with caries prevalence (*F*_(2,199) _= 5.3, *p *< .01). For myopia, the first graders' prevalence rates in the urban and suburban area (29.1% and 24.8% respectively) were significantly higher than in the rural area (16.1%) (both *p *< .01). In addition to myopia, the first graders from the urban area had a significantly lower prevalence rate of untreated dental caries (66.7%) than the participants from the rural area (79.0%) (*p *< .01). Nevertheless, there was no significant difference found among levels of urbanization with regard to obesity prevalence.

**Table 3:** Differences in prevalence rate of health problems by level of urbanization (N = 53,053; Boy = 27,359, Girl = 25,694)

Level of urbanization	Urban		Suburban		Rural		F-value	Scheffe's post hoc test
Health problem	M	SD	M	SD	M	SD		
Myopia	29.1	15.9	24.8	20.3	16.1	16.6	6.1**	R<U, S
Caries	66.7	17.4	71.3	18.2	79.0	21.4	5.3**	U<R
Obesity	8.5	6.2	10.3	6.9	7.4	7.1	1.8	

Statistically significant differences between levels of urbanization: ** indicates p < .01.

### Three top health problems and quantity of medical resources

With respect to the relationship between the three most common health problems (caries, myopia, and obesity) and the quantity of available medical resources, the Pearson's correlation coefficient showed .12 for myopia, -.14 for caries, and -.04 for obesity. No significant association was found between the health problems and the quantity of medical resources.

## Discussion

### General health status

The findings from this study were consistent with the findings from another study on the first-graders in Taipei City, which revealed that 85.1% of students had at least one health problem and, on average, each student had 1.6 health problems [25]. There are few morbidity publications covering solely the first graders to compare in other countries.

### Top three health problems and their association with sex, level of urbanization, and quantity of medical resources

The top three health problems found were dental caries, myopia, and obesity. All three may have long-term effects throughout the lives of first graders.

#### Dental Caries

Dental caries is a major health problem among schoolchildren everywhere in the world. They are the main cause of tooth mortality, dental emergencies, and tooth extraction in this young age. In Taipei County, the top health problem among first graders was untreated dental caries (69.6%). Other studies in Taiwan presented similar trends [26,27]. Also, this caries prevalence is similar to some international studies that focused on children of the same age (i.e., 66.3% for German children [27], 68.4% for India children [28], 71.4% for Mexican children [29], and 70.1% for North Korean children [30]). However, the prevalence of students' untreated caries in Taipei County is higher than in other countries (i.e., 22.0% in America [31,32], and 38.0% in Africa [33]). These findings suggest that the prevalence of caries may not only relate to the country's social-economic status, but also closely relate to lifestyles. As in the more prevalent countries, caries may be due to an increased availability of refined sugar products without a concurrent rise in oral health awareness.

With respect to sex difference, the study results showed the prevalence for boys was higher than for girls. Other studies showed the same conclusion, with boys having higher prevalence of caries than girls [27,34]. One study [26], however, had a reverse finding.

This study demonstrated that the prevalence of untreated caries was significantly different among the levels of urbanization. That is, students who lived in a rural area had the highest prevalence. Different results were found in Africa, where 38.0% of six-year-old children had caries with prevalence being higher in urban rather than rural areas [33]. Although higher prevalence of caries in urban than in rural areas are often reported from developing countries, this does not seem to apply to Taipei County. It is our observation that the children living in all areas of Taipei County were equally exposed to sweets and soft drinks, but the difference was the action to treat the decay. In rural areas, parents tended to ignore the need for treatment for children's caries. They seemed to believe that, because children's teeth would be replaced by permanent teeth, they didn't need to take caries seriously even though the National Health Insurance covered dental treatments free of charge. The untreated caries remained highly prevalent in rural areas and more investigation is needed to determine its behavioral control from parents.

#### Myopia

Literature has suggested that the prevalence of myopia is growing worldwide, and the higher increases were observed in Asia. This study found myopia to rank as the second health problem among first-graders, surpassed only by dental caries. In this study, more than one-fourth (27.1%) of first-graders had myopia. The high prevalence of myopia was similar to some findings reported in East Asia for children at the same age. For instance, prior studies by Saw and colleagues [21,35] found school children's myopia prevalence to be 36.7% for Singapore and 18.5% for Xiamen, China. In contrast with the prevalence of myopia in Australian children (8.4%) [36], the prevalence of myopia found in children from Taipei County and other parts of Asia were a lot higher.

In terms of sex difference, this study showed that girls' prevalence was higher than boys', which is consistent with other studies [18,25,37]. Whether the consequences resulted from genetic or environmental issues still remains unclear.

This study found myopia prevalence to have significant variation among the levels of urbanization. The findings were consistent with the findings from other studies [18,21,38], which revealed significant differences in the prevalence of myopia between residence in urban and rural areas. Children residing in urban areas had a higher prevalence of myopia than children residing in rural areas.

In Taiwan, the myopia prevalence increases by age and occurs more at school ages, especially in the past two decades. Literature shows that the prevalence of myopia has increased from 5.8% to 20.4% between 1983 and 2000 among students in the first-grade, which increased 3.5 fold in the past two decades [38]. The literature also shows that myopia prevalence increased by grade with 20.4% of first-graders, 60.6% of sixth-graders, 80.7% of ninth-graders, and 85% of senior high school students having myopia. In addition, the age at which myopia begins gradually decreased from fifth-grade to second-grade during the period from 1983 to 2000 [38]. In Taiwan, the occurrence of myopia relates to close-to-object studying for longer restless time and watching television screen for learning or entertainment. The phenomena occurred in urban areas more than in rural areas.

#### Obesity

Many studies show obesity relates to lower health-related quality of life [39] and the occurrence of many diseases, including cardiovascular disease, stroke, diabetes [40-45], certain cancers, kidney failure, asthma, arthritis, gallbladder disease, hormonal and reproductive problems, sleep apnea, impaired immune function, blindness [46,47], depression, dementia [48-50], and hypertension [51]. The analysis of large cohorts found that childhood obesity is an important risk factor for adult obesity as 50% of obese children were still obese as adults [52].

Childhood obesity has increased in developed countries. It has become an important health problem of Taiwanese students in the last decade. In Taipei County, obesity was found to be the third health problem in this study, with 9.5% of first-graders being obese. The schoolchildren's prevalence of obesity was consistent with the findings from other studies [27,53] although slightly lower than the prevalence of 13.2% that was found in German children [27].

In term of sex difference, this study revealed that the prevalence of obesity in boys was significantly higher than in girls. This finding was consistent with some relevant studies in Taiwan [25,53,54]. However, some studies [27,55] found no statistical significance. In addition, this study found that the obesity prevalence has no significant difference by the levels of urbanization.

#### Other issues of concern

In SMHI, the parents reported 13.7% of first-graders had at least one diagnosed medical condition, indicating 0.8% of students had heart diseases. However, SEPE found that 1.5% of this population had a heart murmur. This finding showed a sign of possible ignorance or misdiagnosis. In 1998, Liu and colleagues [56] noted that 65.4% of children had been misdiagnosed in terms of heart disease, including 20% of congenital heart disease cases not being diagnosed. Liu's results implied that physical examination can be an important strategy to identify high-risk students. This study also found results similar to the results of a study conducted in Taipei City [25], such as the prevalence of cryptorchidism (2.1% vs. 2.2%), and scoliosis (1.0% vs.0.9%), while these problems were not reported in SMHI. To avoid complex complications and to plan for more efficient treatments, early detection is important, especially for heart disease and cryptorchidism.

This study also found 0.2% of students had asthma through SEPE, which reported the same prevalence as in Taipei City (0.2%) [25]. This prevalence was quite lower than what had been diagnosed (4.1%) from parents' reports and also lower than prevalence reported from the other investigations. For example, Wu and associates had noted that 8.8% of students had asthma in Taipei County [57] and that U.S. children's asthma prevalence ranged 11%–25% [19,58]. SEPE might have an inherent limitation with regard to acute or seasonal diseases like asthma and atopic dermatites.

In addition, this study found no significant relationship between the quantity of medical resources and the three most prevalent health problems (i.e., caries, myopia, and obesity) among the first-graders. It could be due to the coverage of the obligatory national health insurance and the convenient public transportation, which has diminished the discrepancy in medical resources between rural and urban areas.

## Conclusion

The purpose of this study was to assess the health status of first-graders and to examine the relationship between morbidity and sex, level of residence urbanization, and quantity of available medical resources. It is surely startling to find that, amongst 53,053 school children under eight years, only 20.5% of them had no health problem.

The results showed that 79.5% of first-graders had at least one health problem and, on average, each student had about 1.6 health problems. This study also found that there were significant differences in the prevalence between the sub-groups. Generally, the prevalence of health problems for boys was higher than for girls'. The results also indicated that the prevalence of health problems had a significant relationship with residence. In effect, the students who resided in urban areas tended to have higher prevalence of myopia than those who resided in rural areas. However, the opposite was found to be true with regard to the prevalence of dental caries. The first graders' health problems seemed to be closely related to their lifestyle and shaped by their environmental characteristics. Further investigations along this line are suggested.

From the results of this study, we concluded that: First of all, school-based physical examination can be useful to produce a snapshot of the health profile of first-graders and provide insights as to the general health of these school children. Secondly, these results confirmed an evolving epidemic of health problems among first-graders, as evidenced by an increase in the prevalence of dental caries, myopia, and obesity, along with others. Lastly, sex and level of urbanization were associated with the prevalence of caries and myopia at certain levels. More research is suggested as to what level the genetic, environmental, or behavioral factors contribute.

A SEPE prior to school enrollment would help children who suffer from health problems and need treatment. Because students' physical examination is so valuable, it must be done at high quality standards, be offered to all susceptible students, be properly documented, and be explained to their parents. The findings support that the school authorities need to establish a supervisory mechanism to oversee the physical examination programs and to determine whether they meet the quality standards.

It is also suggested that schools prioritize school health programs based on students' health profiles and needs. The findings suggest that, for first-graders, schools in Taipei County that are located in urban areas need to direct their efforts on the prevention of myopia; and those that are located in rural areas need to focus on the prevention of dental caries. In addition, the school administers, school nurses, teachers, health care providers, and parents should work together to improve children's health.

Like other studies, this study has inevitable limitations. Although the existence of disease was identified, the development stage and severity of the diseases were not recorded. Further investigation is suggested to include this information in the examination records. The nature and severity of diseases will allow comparison of studies and is suggested for future investigations. Besides, this study examined the health status differences only based on sex, level of urbanization, and quantity of medical resources. Research that assesses the genetic, environmental, behavioral contribution, and/or other demographic factors (such as single parent family, parent's income, education level, number of siblings, or socioeconomic status) may be included to explain the origins of the health problems. Further investigations on setting the items and diagnostic criteria in physical examination and more discussions on its scientific and medical implications are suggested.

## Abbreviations

SEPE: Student Entry Physical Examination

SMHI: Student Medical History Inventory

ENT: ears-nose-throat

HLA: heart-lung-abdomen

## Competing interests

The author(s) declare that they have no competing interests.

## Authors' contributions

RJY conceived and conducted the study. JJS was responsible for the survey data, statistical analyses, and interpreted the results together with HSC. HLH was responsible for synthesizing analyses. KCL planned and conducted the data transfer. All investigators participated in the writing and revision of the paper.

## Pre-publication history

The pre-publication history for this paper can be accessed here:


